# Reducing Iron Content in Infant Formula from 8 to 2 mg/L Does Not Increase the Risk of Iron Deficiency at 4 or 6 Months of Age: A Randomized Controlled Trial

**DOI:** 10.3390/nu13010003

**Published:** 2020-12-22

**Authors:** Maria Björmsjö, Olle Hernell, Bo Lönnerdal, Staffan K. Berglund

**Affiliations:** 1 Department of Clinical Sciences, Pediatrics, Umeå University, 901 87 Umeå, Sweden; maria.bjormsjo@umu.se (M.B.); olle.hernell@umu.se (O.H.); 2 Department of Nutrition, University of California, Davis, CA 95616, USA; bllonnerdal@ucdavis.edu; 3 Wallenberg Centre for Molecular Medicine (WCMM), Umeå University, 901 87 Umeå, Sweden

**Keywords:** infants, iron supplementation, iron fortification, infant formula, iron status, iron depletion, iron deficiency, iron deficiency anemia, growth, gastrointestinal symptoms

## Abstract

Many infant formulas are fortified with iron at 8–14 mg/L whereas breast milk contains about 0.3 mg/L. Another major difference between breast milk and infant formula is its high concentration of lactoferrin, a bioactive iron-binding protein. The aim of the present study was to investigate how reducing the iron content and adding bovine lactoferrin to infant formula affects iron status, health and development. Swedish healthy full-term formula-fed infants (*n* = 180) were randomized in a double-blind controlled trial. From 6 weeks to 6 months of age, 72 infants received low-iron formula (2 mg/L) fortified with bovine lactoferrin (1.0 g/L) (Lf+), 72 received low-iron formula un-fortified with lactoferrin (Lf−) and 36 received standard formula with 8 mg of iron/L and no lactoferrin fortification as controls (CF). Iron status and prevalence of iron deficiency (ID) were assessed at 4 and 6 months. All iron status indicators were unaffected by lactoferrin. At 4 and 6 months, the geometric means of ferritin for the combined low-iron groups compared to the CF-group were 67.7 vs. 88.7 and 39.5 vs. 50.9 µg/L, respectively (*p* = 0.054 and *p* = 0.056). No significant differences were found for other iron status indicators. In the low-iron group only one infant (0.7%) at 4 months and none at 6 months developed ID. Conclusion: Iron fortification of 2 mg/L is an adequate level during the first half of infancy for healthy term infants in a well-nourished population. Adding lactoferrin does not affect iron status.

## 1. Introduction

Although breast milk is the optimal source of nutrition for infants during the first months of life, a majority of infants worldwide receive partial or complete feeding with infant formula [1]. Efforts to further improve infant formula may provide substantial benefits to infant health. Traditionally, with the intention to avoid iron deficiency (ID) and due to concerns about the bioavailability of iron, many infant formulas are enriched with iron concentrations between 8–14 mg/L, as compared to breast milk, which contains about 0.3 mg/L [2,3]. However, iron excess may have toxic, pro-oxidative effects and contrary to the intention to avoid negative physiological manifestations associated with ID; several studies have reported adverse effects of iron supplementation including impaired growth, changed intestinal microbiome with a shift toward increased growth of pathogens, increased risk of infections and even impaired neurodevelopment [4,5,6,7]. The optimal level of iron in infant formula has rarely been evaluated in clinical trials and there is a need for sufficiently powered randomized controlled studies to better determine the appropriate level of iron in infant formula [2].

Another significant difference between breast milk and infant formula is lactoferrin. Lactoferrin, traditionally not added to infant formula, is an abundant breast milk bioactive protein, believed to be of considerable value to the newborn infant with regard to nutrition, health and development [8]. Lactoferrin is a major iron carrier in human milk but its role in infant iron absorption is unclear. Improved iron status has been observed following bovine lactoferrin supplementation but previous results are divergent [9,10,11,12].

The aim of this project (the LIME study) was to investigate how lowering iron concentration of infant formula from 8 to 2 mg/L and adding 1 g/L of bovine lactoferrin affects health and development of healthy full-term formula-fed Swedish infants. Here, we report the short-term effects on iron status (main outcome), growth and gastrointestinal symptoms.

## 2. Methods

### 2.1. Study Design and Participants

The LIME study is a randomized, double-blind intervention in healthy infants performed between 6 ± 2 weeks and 6 months of age. Full-term Swedish infants (*n* = 252) were recruited, whereof 180 (90 girls and 90 boys) were formula-fed and 72 breastfed (BF) as a reference group (36 girls and 36 boys). All infants were born at Umeå University hospital, Umeå, or the nearby county hospital in Örnsköldsvik, Sweden. Inclusion criteria were birth weight 2500–4500 g, gestational age at birth ≥ 37 weeks, absence of chronic illness and neonatal diagnoses likely to affect any outcome, no previous blood transfusion or iron supplementation and exclusive formula feeding or, for the BF group, exclusive breastfeeding at the inclusion with the intention to exclusively breastfeed until 6 months of age. Eligible infants were identified from delivery records and their parents were contacted by telephone and given oral and written information. To be considered for inclusion, the families had to predetermine whether their infant should be breastfed or given formula. Parents who accepted participation gave written informed consent. The study was approved by the Regional Ethical Review Board in Umeå, ref 2013-245-31M and registered at clinicaltrials.gov, no. NCT02103205.

### 2.2. Intervention

The included formula-fed infants were stratified for gender and assigned randomly in blocks of 10, using computer-generated randomization numbers to receive a formula with low concentration of iron (2 mg/L) and bovine lactoferrin fortification (1.0 g/L) (Lf+, *n* = 72), a formula with low concentration of iron (2 mg/L) without lactoferrin fortification (Lf−, *n* = 72) or the same formula with a higher concentration of iron (8 mg/L) (CF, *n* = 36). In the randomization process, infants in the low-iron formula groups (Lf+ and Lf−) were represented by two groups (each of 36 infants) to prevent identification of group affiliation. The intervention was blinded to both parents and staff until all infants had completed the follow-up at one year of age.

The intervention was from enrollment at 6 ± 2 weeks until 6 months of age. In agreement with World Health Organization (WHO) and national recommendations, parents were instructed to solely feed their infant the study formula or breast milk, respectively, until 4 months of age and were recommended to keep complementary foods to a minimum (taste portions) until 6 months of age. Food and supplements with added pre- or probiotics were not allowed.

The control formula used in the study was a standard formula produced by Mead Johnson Nutrition with adjustment of the iron concentration from 12 to 8 mg/L to match Swedish standard formula recommendations. The Lf+ and Lf− formulas were further modified from this formula by reducing the iron concentration to 2 mg/L, and fortification (Lf+) with 1.0 g/L bovine lactoferrin (Hilmar Ingredients, Hilmar, CA, USA). Formula composition is summarized in Supplemental Table S1.

### 2.3. Data Collection

Infants visited the study center at baseline (6 ± 2 weeks of age) and at 4, 6 and 12 months of age. At each visit, unclothed weight, length and head circumference were collected. Perinatal data including birth weight, length and head circumference, gestational age, Apgar score and neonatal diagnoses were collected from delivery records. Information was obtained from the parents regarding their country of origin, education, smoking habits and the medical history of their infant. Age-adjusted standard deviation scores (SD-scores) were calculated for weight, length and head circumference using WHO growth standards [13,14].

Parents were asked to complete a 3-day food diary every month from 2 to 6 months of age to assess intake of formula and any complementary food. They were instructed to carefully describe, in volume (mL, dL, tea- or tablespoon) or weight (g), the detailed intake of each meal. Daily iron intake from formula and complementary foods, respectively, was calculated, the latter using Dietist Net Pro (Kost och Näringsdata AB, Bromma, Sweden), a dietician tool based on the Swedish Food Agency food composition tables and information from baby food manufacturers.

Infants’ gastrointestinal status and illness were monitored throughout the intervention using a daily record where parents were asked to register each stool and its consistency (hard, soft and watery) as well as a checklist of symptoms (including abdominal pain and medication). The daily stool frequencies were calculated for each infant from the inclusion date to the day of the 6-month visit. Additionally, the longitudinal prevalence (proportion of days) of watery and hard stools, as well as parent-reported abdominal pain and medication were calculated as proxy for abdominal illness. Symptom and gastrointestinal records missing more than 30 days were excluded.

Venous blood samples were drawn at each visit following a minimum of 2 h fasting and 1 h after application of local anesthetic topical cream (combination of Lidocaine 25 mg/g and Prilocaine 25 mg/g). EDTA-treated blood and a serum tube were sent to the local hospital laboratory for direct analyses of hemoglobin (Hb), mean cell volume (MCV), transferrin, iron (Fe) and transferrin saturation (TS). A second tube with serum was, after centrifugation at 1300× *g,* divided into micro tubes and stored frozen at −80 °C until used for analysis at the Pediatric Research lab at Umeå University for ferritin (Thermo Scientific, Waltham, MA, USA), transferrin receptor (TfR) (Ramco Laboratories Inc., Stafford, TX, USA), C-reactive protein (CRP) (R&D Systems Inc., Minneapolis, MN, USA) and hepcidin (Bachem, Peninsula Laboratories, San Carlos, CA, USA). All these analyses were performed in duplicate and samples with a coefficient of variation above 15% or with levels falling outside the measuring range were re-assayed at a different dilution.

Anemia was defined as Hb < 90 g/L at baseline (6 ± 2 weeks) and <105 g/L at 4 months and 6 months. Iron depletion was defined as ferritin < 40 μg/L at inclusion, <20 μg/L at 4 months and <12 μg/L at 6 months. ID was diagnosed when ≥2 of the following indicators of iron status were outside the reference range; ferritin (as defined above), MCV < 73 fL at 4 months and <71 fL at 6 months, TS < 10% (4 and 6 months) and TfR > 11 μg/L (4 and 6 months). Iron deficiency anemia (IDA) was defined as the combination of ID and anemia [2,15,16].

For safety, direct laboratory analyses were assessed by a pediatrician. Two infants developed IDA, one at 4 months and one at 6 months. The former received iron supplementation until 8 months. Both had normalized their iron status at 12 months of age and remained in the study according to the intention to treat principle.

### 2.4. Sample Size and Statistical Analyses

In a pre-study power calculation, the two low-iron groups were sized to a minimum of 64 per group to detect an effect size of 0.5 SD for the lactoferrin intervention. With regard to iron status indicators, we intended to combine the two low-iron groups and compare them to the high-iron group (CF, 8 mg/L). To detect a difference of 20 μg/L in geometric mean ferritin at 6 months using a power of 80% and a significance level of 0.05, group sizes of 129 and 24 infants would be required [17]. To allow for an attrition rate of 10% and to simplify randomization with a 1 to 4 allocation rate, actual group sizes of 144 (72 + 72) and 36 were chosen.

SPSS for Windows version 26.0 (SPSS, Chicago, IL, USA) was used for statistical analyses. For group comparisons of continuous variables, Student’s t test and analysis of variance (ANOVA) with unadjusted post-hoc tests were used. Since ferritin values were non-normally distributed, they were logarithmically transformed and group comparisons are presented as geometric means. Categorical variables were compared using Fisher´s exact test. For other outcomes with skewed distributions (stool frequencies and gastrointestinal symptoms), the Mann–Whitney U test and the Kruskal-Wallis rank-sum test were used.

## 3. Results

### 3.1. Study Group

**Figure 1:**
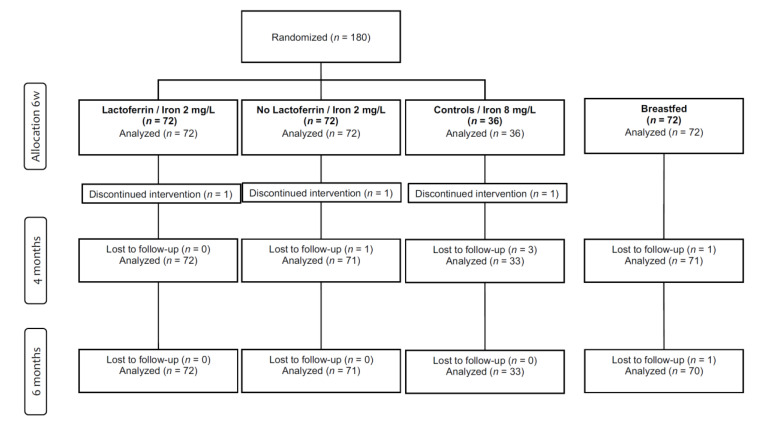
Trial profile. Six infants were lost to follow-up due to early withdrawal (*n* = 2, Lf−, BF), gastrointestinal side effects (*n* = 3, CF) and extensive data collection (*n* = 1, BF). Three infants discontinued intervention due to gastrointestinal symptoms (*n* = 2, CF, LF+) and extensive eczema (*n* = 1, Lf−).

The 252 participants were recruited from June 2014 to June 2018. No infants were excluded and the drop-out rate during intervention was 2% with no group differences (Figure 1). Three participants (1.7%) were considered to be poor compliers since they did not receive the study formula as their main source of nutrition. Two infants (CF, Lf+) switched their formula completely or partially due to gastrointestinal symptoms, and the third infant (Lf−) was temporarily changed to cow milk protein-free formula due to extensive eczema. Background and baseline characteristics are presented in Table 1. There were no significant differences among the formula groups or between the drop-outs and the remaining cases.

### 3.2. Intake of Complementary Food and Iron

Complementary foods, including small taste portions, were introduced to 34%, 35% and 75% of the infants at 4, 5 and 6 months, respectively. Iron intake from formula and complementary foods at each month of intervention is presented in Supplemental Table S2. Mean iron intake from complementary foods was 0.01, 0.05 and 0.25 mg/kg/day at 4, 5 and 6 months, respectively, with no significant differences among the intervention groups.

### 3.3. Iron Status

Blood samples were successfully collected from 98% and 96% of the remaining participants at 4 and 6 months. There were no significant differences in iron status among the formula groups (Table 2). Since iron status was unaffected by adding lactoferrin, the two low-iron groups (Lf+ and Lf−) were combined, according to the original study plan, and compared to the control group (Table 3). A close to significant difference in geometric means of ferritin was observed between the low-iron formula groups and the CF group at both 4 months and 6 months. No significant differences between these groups were found for any of the other iron status indicators including the prevalence of iron depletion, ID and IDA. Infants with CRP level > 8 mg/L were excluded in all iron status assessments. 

### 3.4. Growth and Gastrointestinal Symptoms

No significant differences were found among the formula-fed groups for mean weight, length or head circumferences or their age-corrected SD-scores at 4 or 6 months (Table 4). The BF infants showed significantly lower weight and length compared to the formula-fed groups.

Complete gastrointestinal diaries were collected from 239 infants. No adverse or beneficial effects were observed from adding lactoferrin or the lowered iron concentration (Table 5). The median frequency (IQR, interquartile range) of number of stools per day was 1.3 (1.0–1.8) for the formula-fed infants (*p* = 0.936 for intervention group effect), significantly lower compared to 1.8 (1.2–2.8) per day in the BF group (*p* < 0.001). The Lf− infants reported a significantly higher longitudinal prevalence of hard stools compared to the BF group, a difference not seen in comparison to the Lf+ or CF infants. No differences were seen regarding abdominal pain or use of laxatives, but BF infants reported significantly lower use of Simeticone (2.9%) compared to formula-fed infants (15.9–20.0%) (*p* = 0.007) with no significant intervention effect.

## 4. Discussion

The importance of determining optimal iron levels in infant formula has been emphasized by several authorities due to the possible risks with both low and high-iron intake during the first 6 months of life [2,18,19]. Traditionally, infant formulas are highly fortified with iron due to described risks of ID and assumed lower absorption of iron from formula compared to breast milk [5,20]. The most recent recommendation from the American Academy of Pediatrics (AAP) states that infant formulas should be fortified to 10–12 mg/L [3]. In 2010, the AAP Committee on Nutrition argued that this level is safe with regard to growth, gastrointestinal symptoms and infections. Nevertheless, several recent studies have observed adverse effects of iron excess on neurodevelopment, growth and infections. It has been suggested that the adverse effects interact with baseline iron status and that high-iron intake may be harmful in infants already iron-replete [7,21,22,23,24,25,26]. As a consequence of the reported adverse effects, European authorities keep a more restrictive approach with respect to iron fortification than AAP. Both the European Food Safety Authority (EFSA) and the European Society for Paediatric Gastroenterology Hepatology and Nutrition (ESPGHAN) propose that a level of 2 mg/L is adequate to maintain iron status within the normal range during the first 6 months of life [2,18]. A recent consensus publication from several researchers supported concentrations in the lower range and highlighted the possibility of staging formula iron content by age [19].

Few previous studies have compared different levels of formula iron fortification in infants younger than 6 months [12,27,28,29,30]. Power et al. randomized term, well-nourished and healthy infants to receive formula containing 8.3 mg Fe/100 g (*n* = 74) or 40 mg Fe/100 g (*n* = 75) and observed significantly improved iron status and decreased serum zinc concentrations in the high-iron group but no effects on immune function or incidence of infection [28]. Moffatt et al. randomized 283 infants from very low-income families to receive an iron-fortified formula (12.8 mg/L) or a regular formula (1.1 mg/L) and showed reduced ID and positive, but transient, psychomotor developmental effects in the iron-supplemented formula-fed infants [29]. When comparing infants fed regular iron-containing formula (5 mg Fe/L, *n* = 30) and formula without added iron (<1 mg Fe/L, *n* = 32) during the first 3 months of life, Tuthill et al. found no significant effect on iron status at 3 or 12 months of age [30]. Our study from 2002 is the only previous study exploring the clinical effects of iron levels in the lowest recommended range (2 mg/L). We included 43 infants at 4 ± 2 weeks of age to receive low-iron formula (1.6–2.2 mg/L, *n* = 32 and 4 mg/L, *n* = 11) and concluded that a concentration of 1.6 mg Fe/L was adequate to meet the requirements until 6 months of age [12].

To our knowledge, the present study is the first well-powered study investigating the effect of low-iron concentration formula given to full-term healthy infants before 6 months. Our results strongly suggest that a reduced iron concentration from 8 to 2 mg/L does not increase risk for ID or IDA. There were no cases of ID or IDA at 6 months and only one infant from the low-iron group was diagnosed with ID at 4 months. Notably, both ID and IDA were observed more frequently in the BF reference group, suggesting that the lower iron content of 2 mg/L most likely results in higher iron uptake than in their breastfed peers. We used six different laboratory markers of iron status, all supporting the observation that iron homeostasis was well-balanced even at the lower level of iron fortification. As expected, ferritin concentrations, as a marker of iron stores, decreased slightly more with age in the low-iron groups, but the difference did not reach significance. At 6 months, geometric mean ferritin was 21 μg/L lower in the combined low-iron group. The clinical effects of this difference are most likely low or negligible since the markers reflecting functional iron requirement, e.g., hepcidin and transferrin receptor were not affected.

With regard to added lactoferrin, at least four previous studies have suggested possible improvement in iron status [9,10,11,31]. Chierici et al. found significantly higher serum ferritin levels at day 90 and 150 [11]; King et al. reported significantly higher hematocrit levels but no difference in hemoglobin levels [9] and Ke et al. observed improved iron status markers, including lower prevalence of ID [31]. In contrast to these studies, we did not observe any differences in hematological parameters or iron status among the infants who received formula supplemented with lactoferrin. Our results suggest that in these healthy infants lactoferrin does not affect iron absorption, which is in agreement with our previous study [12]. A possible explanation for the difference among the studies may be the presence of inflammation in enrolled infants. Lactoferrin has anti-inflammatory properties [32] and inflammation also inhibits iron absorption. We speculate that in populations that experience higher levels of inflammation, i.e., due to a higher prevalence of infections, lactoferrin may contribute to iron metabolism through its anti-inflammatory activity [24].

Evaluation of essential functional outcomes is an important key to determining the optimal level of iron fortification [5,7]. Our results showed some differences in growth between the formula-fed and breastfed infants, but no differences among the formula groups, suggesting neither benefits nor harm of the low-iron formula or the added lactoferrin. With regard to gastrointestinal symptoms, there is clinical experience suggesting that iron supplementation can cause side effects, including constipation, even though few studies have assessed this [7]. Furthermore, at least two previous clinical trials with lactoferrin fortification have shown positive gastrointestinal effects such as softer stools [33,34]. We explored several outcomes of gastrointestinal symptoms. The breastfed infants were reported to have more frequent and looser stools and less use of Simeticone compared to the formula-fed groups. However, there were no gastrointestinal side effects related to the intervention in either formula group. Our results strongly suggest that the well-known differences between breastfed and formula-fed infants are not eliminated by reduced iron concentration or lactoferrin supplementation to infant formula.

The major strengths of the present study are its randomized design, low dropout rate and the very high compliance to the intervention. It was set up in a well-organized pediatric nutritional research center providing good conditions for recruitment and follow-up. The study population consisted of healthy full-term infants living, from a global perspective, under privileged circumstances regarding socioeconomic and disease burden. This background homogeneity brings strengths but also limits the applicability to other populations. Other limitations were that even though this is a relatively large randomized trial, the power is still too low for detecting small differences in iron status and particularly rates of ID and IDA.

## 5. Conclusions

Reducing infant formula iron concentration from 8 to 2 mg/L marginally reduced iron stores but did not increase the risk of ID or IDA at 4 or 6 months of age. Additionally, adding lactoferrin had no effect on iron status and no benefits or adverse effects were observed on growth or gastrointestinal symptoms. Consequently, the study supports the conclusion that 2 mg/L is an adequate and safe level of iron fortification in a well-nourished population with low risk of ID, and that it results in an iron status more similar to breastfed infants. Future evaluations of morbidity and neurodevelopment will be performed to further compare benefits and harm of the different levels of iron fortification and lactoferrin supplementation.

## Figures and Tables

**Table 1:** Background and baseline characteristics.

	Lf+	Lf−	CF	*p*-Value	BF
**Background**	*n* = 72	*n* = 72	*n* = 36		*n* = 72
Female	36 (50)	36 (50)	18 (50)	0.503	36 (50)
Gestational age, weeks	39.6 ± 1.2	39.8 ± 1.2	39.5 ± 1.3	0.316	39.8 ± 1.2
Vaginal delivery	52 (72)	57 (79)	28 (78)	0.599	62 (86)
Apgar score at 5 min	9.3 ± 0.8	9.3 ± 0.9	9.3 ± 0.9	>0.999	9.1 ± 0.8
Birth weight, kg	3.59 ± 0.44	3.54 ± 0.43	3.50 ± 0.37	0.549	3.54 ± 0.36
Birth length, cm	50.1 ± 1.8	50.0 ± 1.8	50.3 ± 1.4	0.689	50.3 ± 1.9
Head circumference, cm	35.2 ± 1.5	35.0 ± 1.3	34.8 ± 1.3	0.400	35.1 ± 1.0
Neonatal unit care	4 (5.6)	5 (6.9)	3 (8.3)	0.926	2 (2.8)
Antibiotic treatment day 0–7	0	0	0		0
Maternal age, years	29.6 ± 4.1	30.0 ± 5.4	29.7 ± 4.7	0.877	30.8 ± 4.8
Multiple gestation	47 (65.3)	42 (58.3)	18 (50.0)	0.303	38 (52.8)
Mother of non-Nordic descent	1 (1.4)	4 (5.6)	1 (2.8)	0.446	3 (4.2)
Maternal education at university	30 (41.7) *	29 (40.3) *	15 (41.7) *	0.983	49 (68.1)
Maternal smoking during pregnancy	4 (5.6)	3 (4.2)	1 (2.8)	0.904	0
Iron supplementation during pregnancy	55 (76.4)	54 (75.0)	28 (77.8)	0.948	55 (76.4)
**Baseline**					
Weight, kg	5.09 ± 0.49	5.04 ± 0.59	5.02 ± 0.54	0.737	5.10 ± 0.56
Length, cm	56.4 ± 1.7	56.1 ± 1.7	56.1 ± 1.7	0.605	56.6 ± 1.9
Head circumference, cm	38.8 ± 1.2	38.6 ± 1.2	38.4 ± 0.9	0.186	38.7 ± 1.0
Hemoglobin, g/L	112 ± 12	115 ± 14	114 ± 13	0.398	118 ± 13
Mean cell volume, fL	89.6 ± 3.3	89.0 ± 3.1	88.4 ± 3.9	0.232	88.5 ± 3.4
Ferritin, μg/L	339 ± 154 *	312 ± 157 *	345 ± 226	0.564	399 ± 185
Ferritin, geometric mean, μg/L	304 ± 1.65	277 ± 1.66	284 ± 1.91	0.600	349 ± 1.84
Transferrin, g/L	1.81 ± 0.28	1.79 ± 0.25	1.82 ± 0.22	0.760	1.86 ± 0.27
Transferrin saturation, %	37.5 ± 10.5	37.8 ± 11.9	35.7 ± 9.7	0.617	34.0 ± 11.7
Transferrin receptor, mg/L	2.86 ± 0.90	2.84 ± 0.77	2.92 ± 0.71	0.892	3.18 ± 0.92
Hepcidin, ng/mL	96.4 ± 35.0	93.3 ± 36.1	93.9 ± 40.5	0.876	81.5 ± 30.4

Values are presented as mean ± SD or number (%). *p*-values for differences among intervention groups using analysis of variance for means and Fisher´s exact test for proportions. * Significantly different from BF infants (*p* < 0.05). Lf+ = infants receiving low-iron formula (2 mg/L) fortified with bovine lactoferrin (1.0 g/L). LF− = infants receiving low-iron formula (2 mg/L) without lactoferrin fortification. CF = infants receiving standard formula with normal iron concentration (8 mg/L) and without lactoferrin fortification. BF = breastfed infants.

**Table 2:** Iron status at 4 and 6 months in 180 formula-fed infants randomized into three different infant formulas compared to a breastfed reference group.

	Lf+	Lf−	CF	*p*-Value	BF
**4 Months**	*n* = 63–69	*n* = 67–68	*n* = 31–32		*n* = 65–69
Hemoglobin, g/L	113.0 ± 6.7	113.9 ± 6.6	114.3 ± 8.2	0.599	114.7 ± 7.6
Mean cell volume, fL	80.0 ± 3.1 *	79.5 ± 3.1	79.0 ± 4.1	0.378	78.4 ± 3.2
Ferritin, μg/L	87.2 ± 58.4 *	85.4 ± 63.1 *	105.1 ± 65.4	0.310	123.2 ± 86.6
Ferritin, geometric mean, μg/L	69.28 ± 2.04 *	66.18 ± 2.08 *	88.70 ± 1.82	0.148	91.68 ± 2.39
Iron, μmol/L	9.38 ± 2.31	9.88 ± 3.15	10.59 ± 2.60	0.121	9.07 ± 2.35
Transferrin, g/L	2.32 ± 0.37	2.29 ± 0.27	2.31 ± 0.28	0.840	2.32 ± 0.40
Transferrin saturation, %	16.5 ± 4.8	17.8 ± 6.4	18.4 ± 4.7	0.202	15.9 ± 5.1
Transferrin receptor, mg/L	5.37 ± 0.98	5.32 ± 1.17	5.42 ± 0.96	0.907	5.43 ± 1.37
Hepcidin, ng/mL	50.3 ± 29.0	50.2 ± 26.9	53.8 ± 20.9	0.801	40.9 ± 27.2
Iron depletion	3 (4.3)	2 (2.9)	0	0.842	4 (5.9)
Iron deficiency	1 (1.4)	0	0	>0.999	3 (4.4)
Iron deficiency anemia	0	0	0	NA	1 (1.4)
**6 Months**	*n* = 65–67	*n* = 64–67	*n* = 29–32		*n* = 65–67
Hemoglobin, g/L	115.4 ± 6.4	116.5 ± 8.6	116.6 ± 6.6	0.626	115.2 ± 7.7
Mean cell volume, fL	76.5 ± 2.7	76.5 ± 2.4	76.2 ± 4.0	0.822	75.4 ± 3.2
Ferritin, μg/L	50.9 ± 35.0	47.1 ± 33.6	59.3 ± 34.7	0.286	62.2 ± 53.8
Ferritin, geometric mean, μg/L	40.71 ± 1.98	38.41 ± 1.89	50.94 ± 1.75	0.142	44.23 ± 2.33
Iron, μmol/L	9.46 ± 2.95	9.64 ± 2.71	9.31 ± 3.65	0.864	8.69 ± 6.10
Transferrin, g/L	2.36 ± 0.33	2.39 ± 0.29	2.31 ± 0.24	0.478	2.44 ± 0.37
Transferrin saturation, %	16.3 ± 5.5	16.5 ± 5.3	16.1 ± 6.2	0.958	14.2 ± 8.5
Transferrin receptor, mg/L	5.12 ± 1.11	5.42 ± 1.32	5.08 ± 0.98	0.243	5.36 ± 1.35
Hepcidin, ng/mL	45.1 ± 22.9 *	41.4 ± 21.4 *	48.7 ± 18.4 *	0.286	31.6 ± 27.0
Iron depletion	2 (3.0)	2 (3.0)	0	>0.999	5 (7.6)
Iron deficiency	0	0	0	NA	7 (10.6)
Iron deficiency anemia	0	0	0	NA	1 (1.5)

Values are presented as mean ± SD or number (%). *p*-values for differences among intervention groups using analysis of variance for means and Fisher´s exact test for proportions. * Significantly different from BF infants (*p* < 0.05). Infants with C-reactive protein level > 8 mg/L were excluded.

**Table 3:** Iron status at 4 and 6 months in formula-fed infants randomized to a low-iron formula (2 mg/L) compared controls (8 mg/L).

	Low Iron (2 mg/L)	CF (8 mg/L)	*p*-Value
**4 Months**	*n* = 130–137	*n* = 31–32	
Hemoglobin, g/L	113.4 ± 6.6	114.3 ± 8.2	0.526
Mean cell volume, fL	79.7 ± 3.1 *	79.0 ± 4.1	0.242
Ferritin, μg/L	86.3 ± 60.7 *	105.1 ± 65.4	0.127
Ferritin, geometric mean, μg/L	67.67 ± 2.06	88.70 ± 1.82	0.054
Iron, μmol/L	9.63 ± 2.76	10.59 ± 2.60	0.079
Transferrin, g/L	2.30 ± 0.32	2.31 ± 0.28	0.939
Transferrin saturation, %	17.2 ± 5.7	18.4 ± 4.7	0.274
Transferrin receptor, mg/L	5.35 ± 1.08	5.42 ± 0.96	0.721
Hepcidin, ng/mL	50.3 ± 27.9 *	53.8 ± 20.9	0.506
**6 Months**	*n* = 131–134	*n* = 29–32	
Hemoglobin, g/L	116.0 ± 7.6	116.6 ± 6.6	0.641
Mean cell volume, fL	76.5 ± 2.5	76.2 ± 4.0	0.635
Ferritin, μg/L	49.0 ± 34.2	59.3 ± 34.7	0.147
Ferritin, geometric mean, μg/L	39.53 ± 1.93	50.94 ± 1.75	0.056
Iron, μmol/L	9.55 ± 2.83	9.31 ± 3.65	0.685
Transferrin, g/L	2.37 ± 0.31	2.31 ± 0.24	0.273
Transferrin saturation, %	16.4 ± 5.4	16.1 ± 6.2	0.830
Transferrin receptor, mg/L	5.27 ± 1.22	5.08 ± 0.98	0.408
Hepcidin, ng/mL	43.3 ± 22.2 *	48.7 ± 18.4 *	0.211

Values are presented as mean ± SD or number (%). *p*-values for differences between controls and the two low-iron groups combined using Student’s *t*-test. * Significantly different from BF infants (*p* < 0.05). Infants with C-reactive protein level > 8 mg/L were excluded.

**Table 4:** Anthropometric data at 4 and 6 months in 180 formula-fed infants randomized into three different infant formulas compared to a breastfed reference group.

	Lf+	Lf−	CF	*p*-Value	BF
**4 Months**	*n* = 72	*n* = 71	*n* = 33		*n* = 71
Weight, kg	6.98 ± 0.75	7.00 ± 0.79	7.05 ± 0.82	0.910	6.76 ± 0.78
Length, cm	63.3 ± 1.9	63.3 ± 1.8	63.1 ± 2.2	0.840	63.0 ± 2.2
Head circumference, cm	41.9 ± 1.2	41.7 ± 1.1	41.5 ± 0.9	0.315	41.5 ± 1.1
Weight SD score	0.31 ± 0.82	0.34 ± 0.88	0.40 ± 0.87	0.878	0.04 ± 0.92
Length SD score	0.18 ± 0.89	0.16 ± 0.82	0.08 ± 0.92	0.871	0.04 ± 0.96
Head circumference SD score	0.65 + 0.89	0.52 ± 0.82	0.38 ± 0.63	0.258	0.35 ± 0.85
**6 Months**	*n* = 72	*n* = 71	*n* = 33		*n* = 70
Weight, kg	8.14 ± 0.80 *	8.19 ± 0.98 *	8.29 ± 0.91 *	0.739	7.78 ± 0.85
Length, cm	67.5 ± 2.1 *	67.2 ± 2.0 *	67.2 ± 2.3 *	0.646	66.3 ± 2.3
Head circumference, cm	43.7 ± 1.3	43.5 ± 1.3	43.5 ± 0.9	0.517	43.3 ± 1.1
Weight SD score	0.55 ± 0.77 *	0.60 ± 0.94 *	0.71 ± 0.85 *	0.684	0.17 ± 0.92
Length SD score	0.39 ± 0.91 *	0.26 ± 0.82 *	0.28 ± 0.95 *	0.655	-0.12 ± 1.01
Head circumference SD score	0.77 ± 0.92	0.64 ± 0.88	0.56 ± 0.68	0.453	0.42 ± 0.83
Weight gain, g/day 6w-6mo	23.2 ± 4.2 *	23.4 ± 4.8 *	24.6 ± 4.9 *	0.310	20.6 ± 4.6
Length gain, mm/day 6w-6mo	0.84 ± 0.10 *	0.83 ± 0.08 *	0.84 ± 0.10 *	0.566	0.75 ± 0.09
Head circumference gain, mm/day 6w-6mo	0.37 ± 0.05 *	0.37 ± 0.04 *	0.38 ± 0.06 *	0.358	0.35 ± 0.04

Values are presented as mean ± SD. *p*-values for differences among intervention groups using analysis of variance for means. * Significantly different from BF infants (*p* < 0.05).

**Table 5:** Gastrointestinal symptoms in 180 formula-fed infants randomized into three different infant formulas compared to a breastfed reference group.

	Lf+	Lf−	CF	*p*-Value	BF
**Daily Frequency**	*n* = 70	*n* = 69	*n* = 32		*n* = 68
Stools per day, *n*	1.36 (1.0;1.9) *	1.32 (1.0;1.7) *	1.22 (1.0;1.8) *	0.936	1.77 (1.2;2.8)
Watery stools per day, *n*	0.05 (0.0;0.2)	0.05 (0.0;0.2)	0.03 (0.0;0.2)	0.640	0.02 (0.0;0.1)
Soft stools per day, *n*	1.2 (0.8;1.6) *	1.2 (0.9;1.5) *	1.1 (0.9;1.6) *	0.884	1.57 (0.8;2.5)
Hard stools per day, *n*	0.0 (0.0;0.0)	0.0 (0.0;0.0) *	0.0 (0.0;0.0)	0.530	0.00 (0.0;0.0)
**Longitudinal Prevalence**					
Days with watery stools, %	0.8 (0.8;12.8)	3.7 (0.0;10.6)	2.8 (0.0;9.2)	0.598	0.8 (0.0;6.7)
Days with hard stools, %	0.0 (0.0;0.6)	0.0 (0.0;0.8) *	0.0 (0.0;0.0)	0.554	0.0 (0.0;0.0)
Days with abdominal pain, %	0.7 (0.0;3.0)	2.0 (0.0;2.4)	0.0 (0.0;4.4)	0.543	0.0 (0.0;0.8)
**Use of GI Medication**					
Any use of oral laxative	1 (1.4)	2 (2.9)	3 (9.4)	0.138	1 (1.5)
Any use of Simeticone	14 (20.0) *	11 (15.9) *	6 (18.8) *	0.840	2 (2.9)

Values are presented as median (25th; 75th percentile) or number (%). *p*-values for differences among intervention groups using Kruskal-Wallis one-way test for medians and Fisher’s exact test for proportions. * Significantly different from BF infants (*p* < 0.05).

## Data Availability

The data presented in this study are available on request from the corresponding author.

## Supplementary Materials

The following are available online at https://www.mdpi.com/2072-6643/13/1/3/s1, Table S1: Study formula composition, Table S2: Dietary intake according to 3-day dietary diary during the infant formula intervention.
