# Supplementary material for: Reducing Iron Content in Infant Formula from 8 to 2 mg/L Does Not Increase the Risk of Iron Deficiency at 4 or 6 Months of Age: A Randomized Controlled Trial
Source: Nutrients. 2020 Dec 22;13(1):3. doi: 10.3390/nu13010003 (PMC7821997; doi:10.3390/nu13010003)
Supplement: Supplementary file 1 [file nutrients-13-00003-s001.zip › Supplemental table S1.pdf]

Reducing iron content in infant formula from 8 to 2 mg/L does not increase the risk of iron deficiency at 4 or 6 months of age: a randomized controlled trial

Maria Björmsjö[1], MD, Olle Hernell[1], MD, PhD, Bo Lönnerdal[2], PhD, Staffan K Berglund[1,3*],MD, PhD

Supplementary Material

Supplemental Table S1. Study formula composition.

NUTRIENT per 100 ml	Lf-	Lf+	CF
Energy, kcal	68	68	68
Protein, g	1.4	1.4	1.4
Including Lactoferrin, g	0	0.1	0
Fat, g	3.7	3.7	3.7
Total carbohydrates*, g	7.4	7.4	7.4
Polydextrose (PDX) , g	0.2	0.2	0.2
Vitamin A - Total, IU	204	204	204
Vit D (cholecalciferol), IU	42	42	42
Vitamin E - Total, IU	1.2	1.2	1.2
Vitamin K, µg	5	5	5
Vitamin B1 (thiamin), µg	54	54	54
Vitamin B2 (riboflavin), µg	122	122	122
Vitamin B6 (pyridoxine), µg	41	41	41
Vitamin B12, µg	0.18	0.18	0.18
Niacin (vitamin B3), µg	544	544	544
Folic Acid - Total, mg	11	11	11
Vitamin C, mg	14	14	14
Calcium, mg	53	53	53
Phosphorus, mg	29	29	29
Magnesium, mg	5	5	5
Iron, mg	0.2	0.2	0.8
Zinc, mg	0.7	0.7	0.7
Sodium, mg	20	20	20
Potassium, mg	82	82	82
Chloride, mg	48	48	48

* Including a 0.4 g blend of polydextrose (PDX) and galacto-oligosaccharides (GOS) in 1:1 ratio
