# Supplementary material for: Reducing Iron Content in Infant Formula from 8 to 2 mg/L Does Not Increase the Risk of Iron Deficiency at 4 or 6 Months of Age: A Randomized Controlled Trial
Source: Nutrients. 2020 Dec 22;13(1):3. doi: 10.3390/nu13010003 (PMC7821997; doi:10.3390/nu13010003)
Supplement: Supplementary file 1 [file nutrients-13-00003-s001.zip › Supplemental table S2.pdf]

Reducing iron content in infant formula from 8 to 2 mg/L does not increase the risk of iron deficiency at 4 or 6 months of age: a randomized controlled trial

Maria Björmsjö[1], MD, Olle Hernell[1], MD, PhD, Bo Lönnerdal[2], PhD, Staffan K Berglund[1,3*],MD, PhD

Supplementary Material

Supplemental Table S2. Dietary intake according to 3-day dietary diary during the infant formula intervention.

	Lf+	Lf-	CF	p-value	BF
**Formula intake per day, ml**					
2 months	808±150	816±133	823±135	.88	N/A
3 months	868±164	836±133	857±149	.46	N/A
4 months	908±148	889±149	955±138	.12	N/A
5 months	945±204	898±173	896±166	.27	N/A
6 months	781±262	764±227	669±326	.22	N/A
**Iron intake from infant formula, mg/kg/day**					
2 months	0.29±0.05	0.30±0.05	1.21±0.20	< .001	N/A
3 months	0.28±0.05	0.27±0.04	1.09±0.19	< .001	N/A
4 months	0.26±0.04	0.26±0.04	1.09±0.15	< .001	N/A
5 months	0.25±0.05	0.24±0.04	0.94±0.18	< .001	N/A
6 months	0.19±0.06	0.19±0.06	0.65±0.32	< .001	N/A
**Iron intake from complementary foods, mg/kg/day**					
4 months	0.00±0.01	0.00±0.01	0.02±0.13	.13	0.01±0.03
5 months	0.02±0.06	0.04±0.08	0.05±0.11	.21	0.05±0.12
6 months	0.26±0.31	0.21±0.35	0.31±0.32	.41	0.17±0.29

Values are presented as mean ± SD.

Infants partly fed another formula than the study formula were excluded (4 at 2 mo, 3 at 3 mo, 4 at 4 mo, 3 at 5 mo, 10 at 6 mo)
